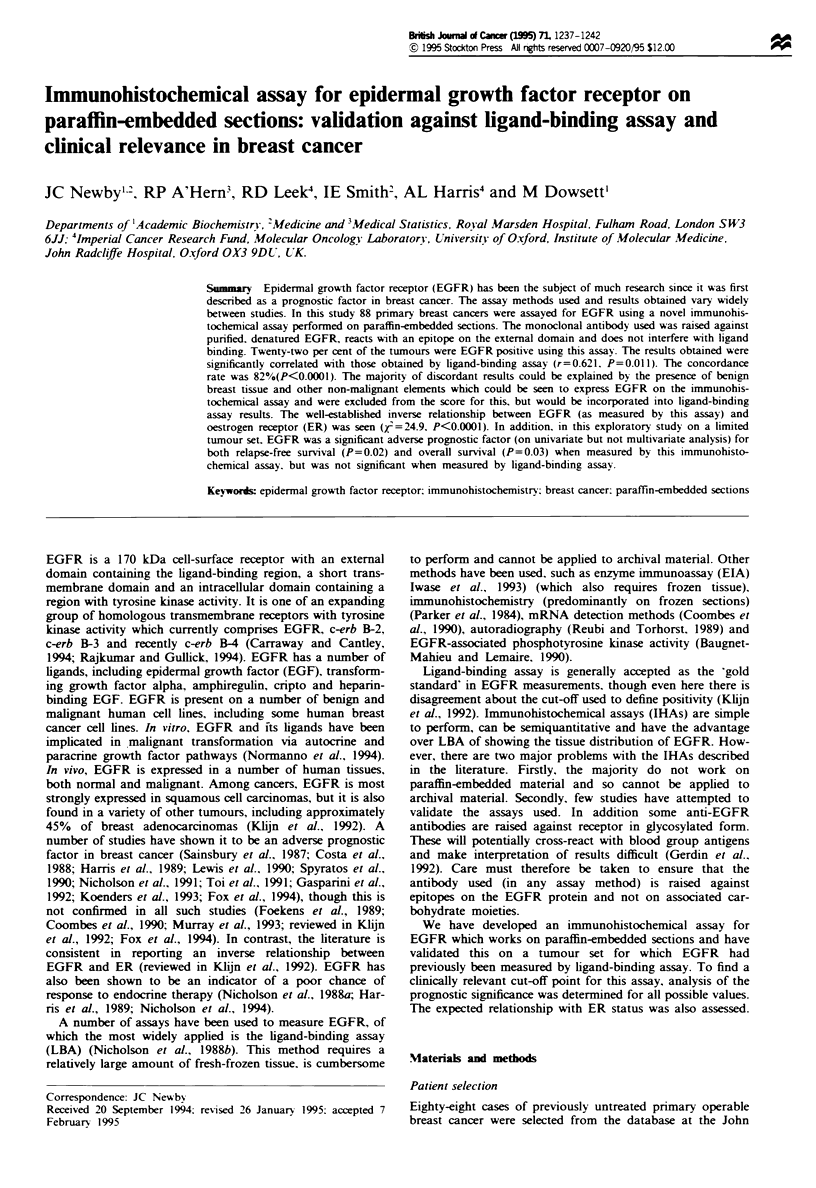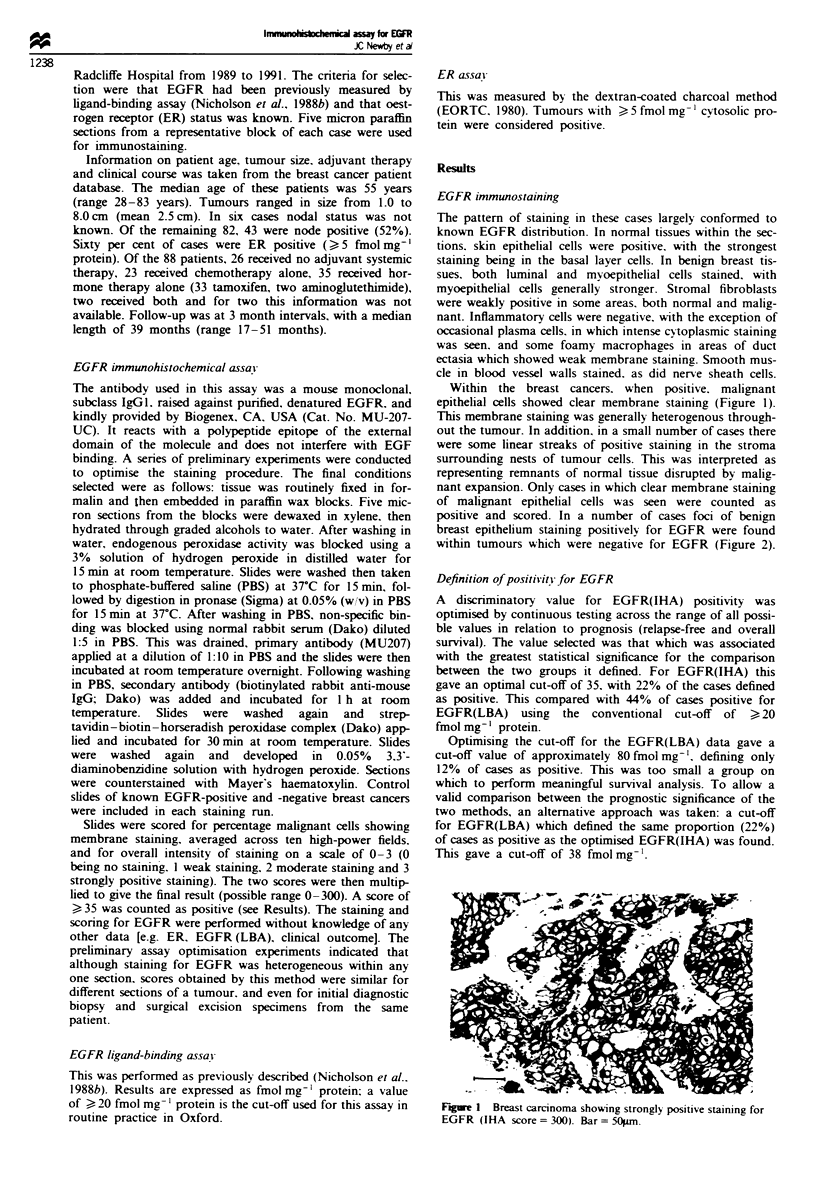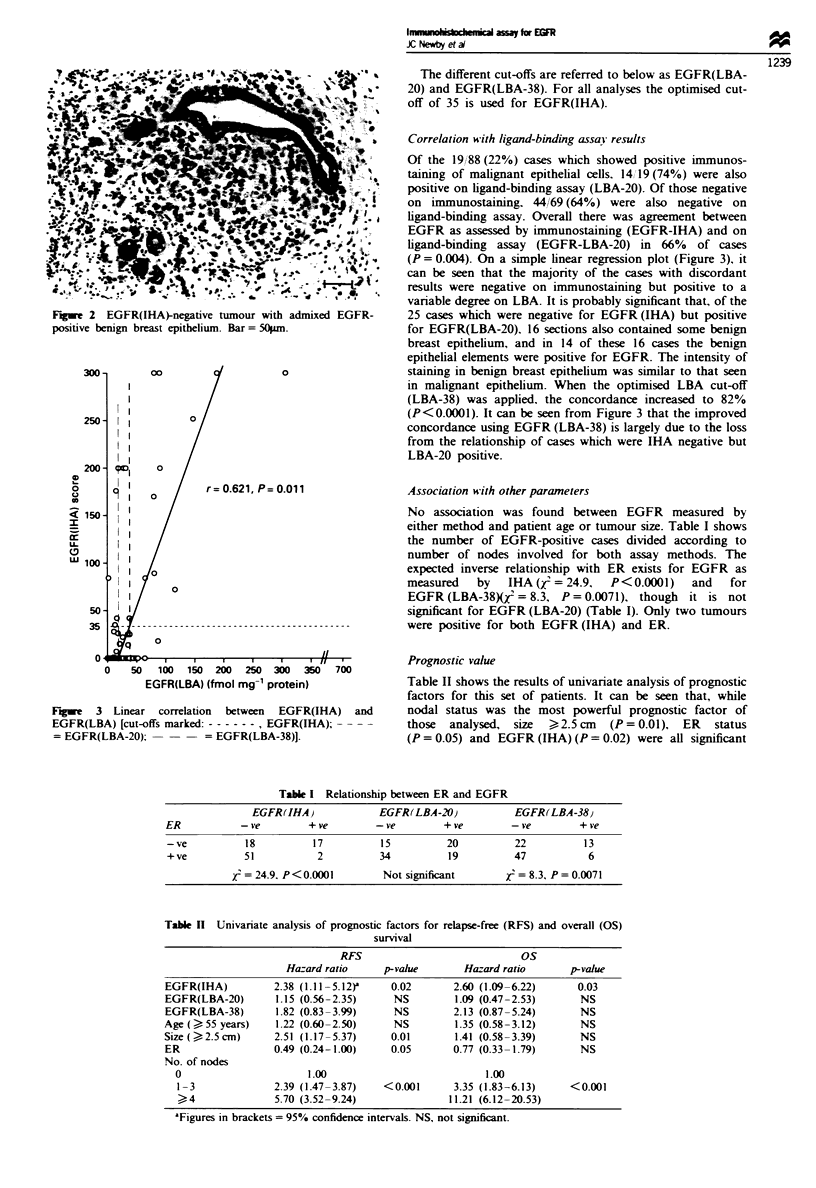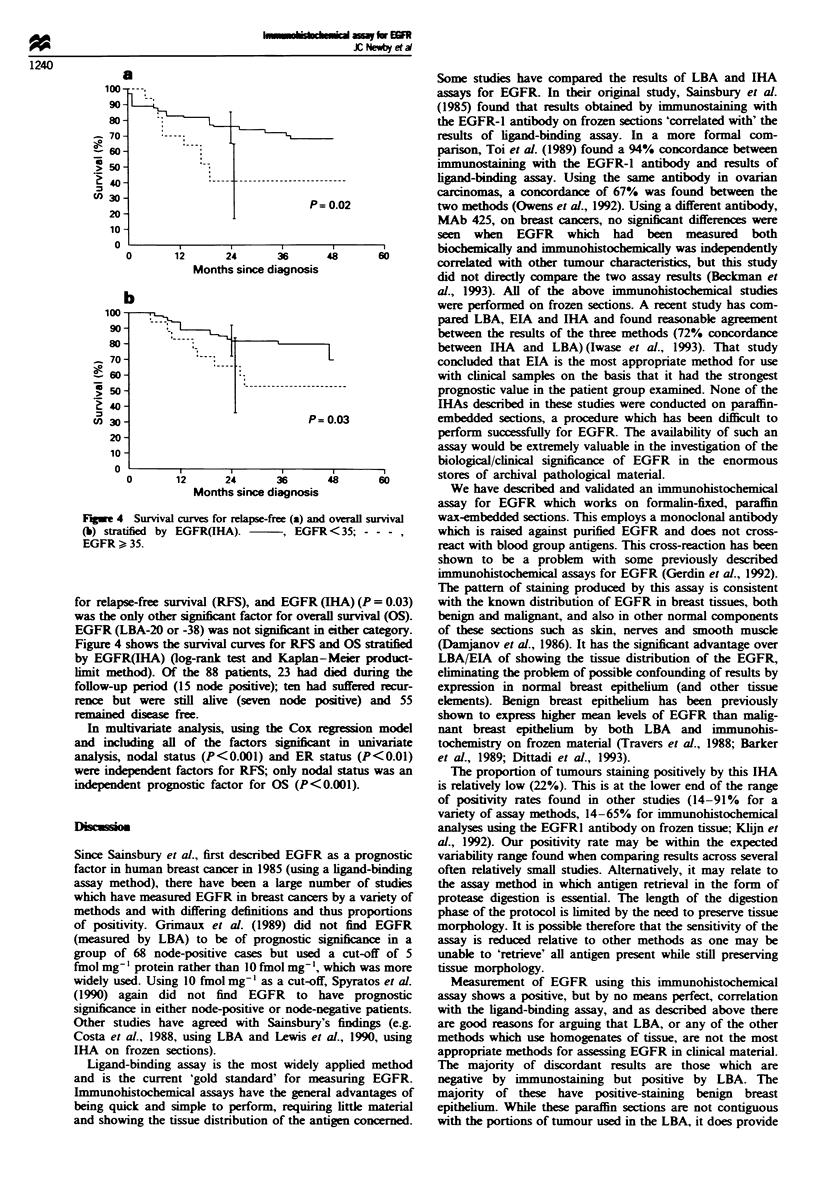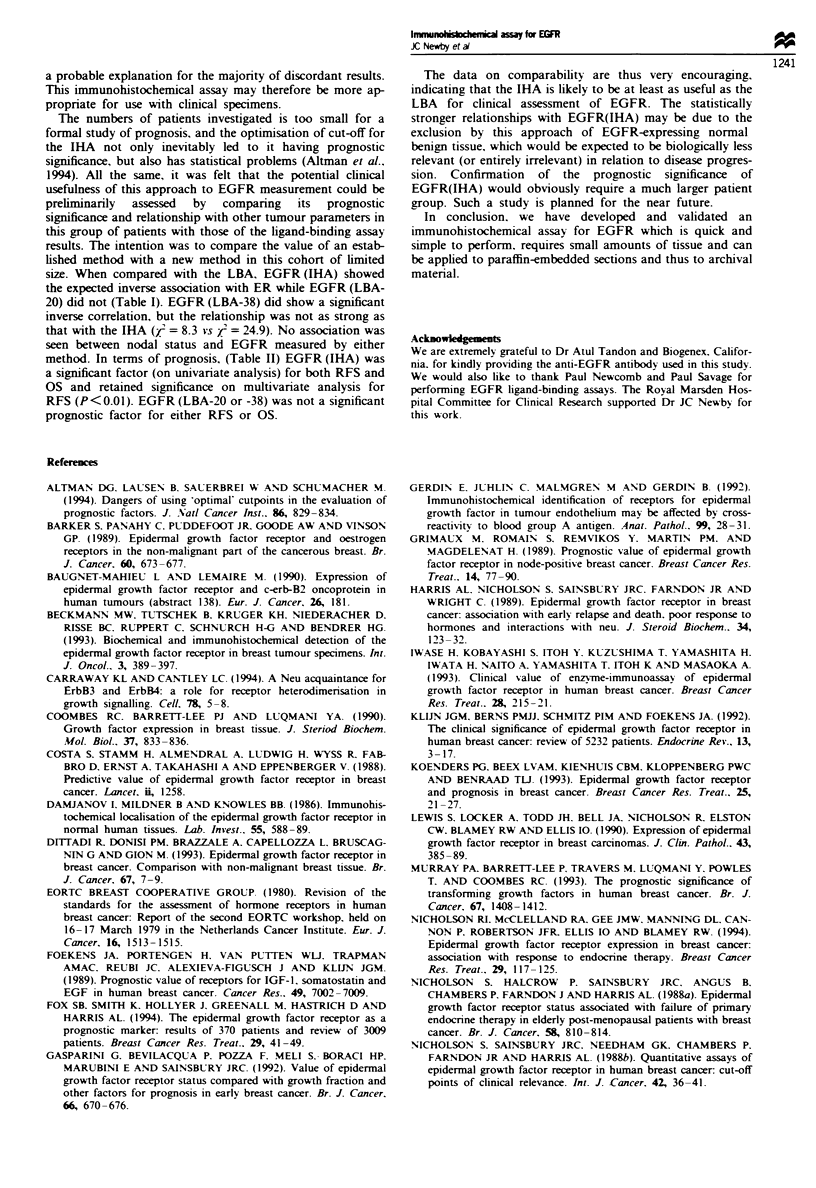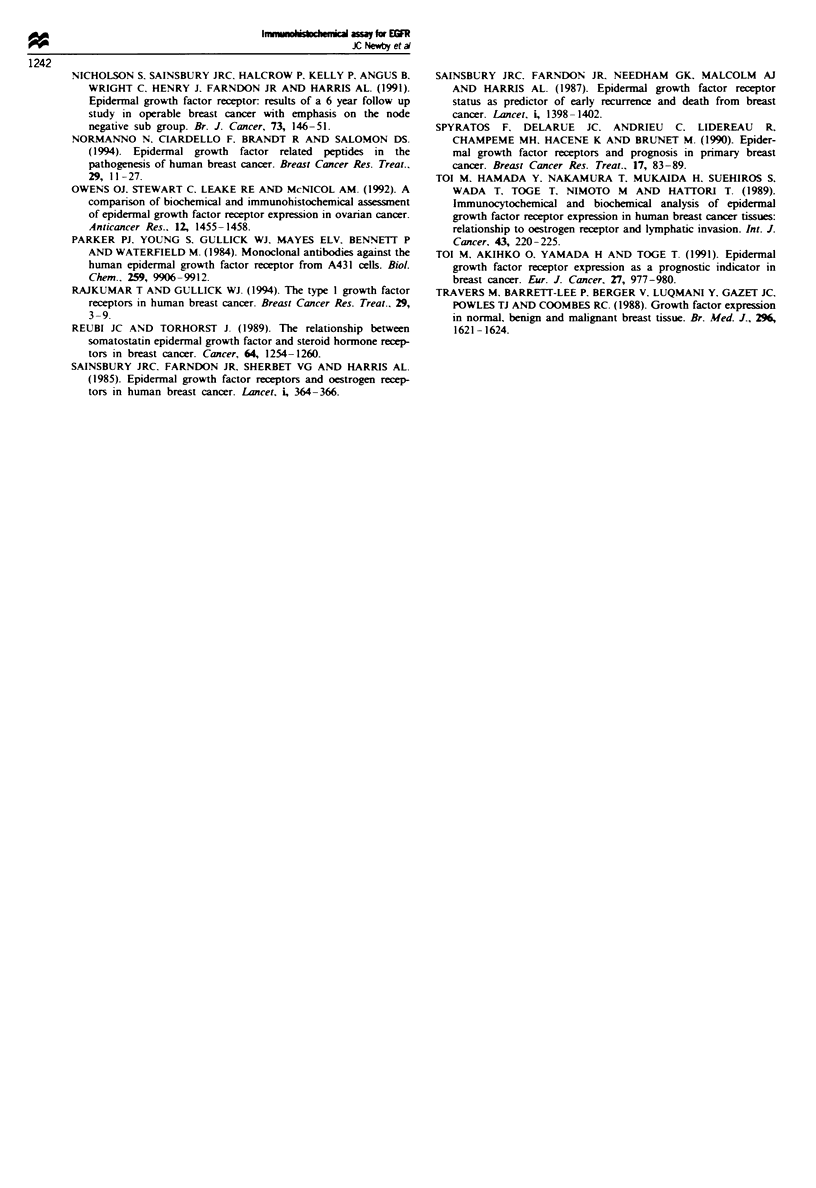# Immunohistochemical assay for epidermal growth factor receptor on paraffin-embedded sections: validation against ligand-binding assay and clinical relevance in breast cancer.

**DOI:** 10.1038/bjc.1995.239

**Published:** 1995-06

**Authors:** J. C. Newby, R. P. A'Hern, R. D. Leek, I. E. Smith, A. L. Harris, M. Dowsett

**Affiliations:** Department of Academic Biochemistry, Royal Marsden Hospital, London, UK.

## Abstract

**Images:**


British Journal of Cancer (1995) 71, 1237-1242

© 1995 Stockton Press All rights reserved 0007-0920/95 $12.00

### Immunohistochemical assay for epidermal growth factor receptor on paraffin-embedded sections: validation against ligand-binding assay and clinical relevance in breast cancer

JC Newby[1,2], RP A'Hern[3], RD Leek[4], IE Smith[2], AL Harris[4] and M Dowsett[1]

*Departments of [1]Academic Biochemistry, [2]Medicine and [3]Medical Statistics, Royal Marsden Hospital, Fulham Road, London SW3 6JJ; [4]Imperial Cancer Research Fund, Molecular Oncology Laboratory, University of Oxford, Institute of Molecular Medicine, John Radcliffe Hospital, Oxford OX3 9DU, UK.*

**Summary** Epidermal growth factor receptor (EGFR) has been the subject of much research since it was first described as a prognostic factor in breast cancer. The assay methods used and results obtained vary widely between studies. In this study 88 primary breast cancers were assayed for EGFR using a novel immunohistochemical assay performed on paraffin-embedded sections. The monoclonal antibody used was raised against purified, denatured EGFR, reacts with an epitope on the external domain and does not interfere with ligand binding. Twenty-two per cent of the tumours were EGFR positive using this assay. The results obtained were significantly correlated with those obtained by ligand-binding assay ($r=0.621$, $P=0.011$). The concordance rate was 82%($P<0.0001$). The majority of discordant results could be explained by the presence of benign breast tissue and other non-malignant elements which could be seen to express EGFR on the immunohistochemical assay and were excluded from the score for this, but would be incorporated into ligand-binding assay results. The well-established inverse relationship between EGFR (as measured by this assay) and oestrogen receptor (ER) was seen ($\chi^2=24.9$, $P<0.0001$). In addition, in this exploratory study on a limited tumour set, EGFR was a significant adverse prognostic factor (on univariate but not multivariate analysis) for both relapse-free survival ($P=0.02$) and overall survival ($P=0.03$) when measured by this immunohistochemical assay, but was not significant when measured by ligand-binding assay.

**Keywords:** epidermal growth factor receptor; immunohistochemistry; breast cancer; paraffin-embedded sections

EGFR is a 170 kDa cell-surface receptor with an external domain containing the ligand-binding region, a short transmembrane domain and an intracellular domain containing a region with tyrosine kinase activity. It is one of an expanding group of homologous transmembrane receptors with tyrosine kinase activity which currently comprises EGFR, c-*erb* B-2, c-*erb* B-3 and recently c-*erb* B-4 (Carraway and Cantley, 1994; Rajkumar and Gullick, 1994). EGFR has a number of ligands, including epidermal growth factor (EGF), transforming growth factor alpha, amphiregulin, cripto and heparin-binding EGF. EGFR is present on a number of benign and malignant human cell lines, including some human breast cancer cell lines. *In vitro*, EGFR and its ligands have been implicated in malignant transformation via autocrine and paracrine growth factor pathways (Normanno *et al.*, 1994). *In vivo*, EGFR is expressed in a number of human tissues, both normal and malignant. Among cancers, EGFR is most strongly expressed in squamous cell carcinomas, but it is also found in a variety of other tumours, including approximately 45% of breast adenocarcinomas (Klijn *et al.*, 1992). A number of studies have shown it to be an adverse prognostic factor in breast cancer (Sainsbury *et al.*, 1987; Costa *et al.*, 1988; Harris *et al.*, 1989; Lewis *et al.*, 1990; Spyratos *et al.*, 1990; Nicholson *et al.*, 1991; Toi *et al.*, 1991; Gasparini *et al.*, 1992; Koenders *et al.*, 1993; Fox *et al.*, 1994), though this is not confirmed in all such studies (Foekens *et al.*, 1989; Coombes *et al.*, 1990; Murray *et al.*, 1993; reviewed in Klijn *et al.*, 1992; Fox *et al.*, 1994). In contrast, the literature is consistent in reporting an inverse relationship between EGFR and ER (reviewed in Klijn *et al.*, 1992). EGFR has also been shown to be an indicator of a poor chance of response to endocrine therapy (Nicholson *et al.*, 1988*a*; Harris *et al.*, 1989; Nicholson *et al.*, 1994).

A number of assays have been used to measure EGFR, of which the most widely applied is the ligand-binding assay (LBA) (Nicholson *et al.*, 1988*b*). This method requires a relatively large amount of fresh-frozen tissue, is cumbersome to perform and cannot be applied to archival material. Other methods have been used, such as enzyme immunoassay (EIA) Iwase *et al.*, 1993) (which also requires frozen tissue), immunohistochemistry (predominantly on frozen sections) (Parker *et al.*, 1984), mRNA detection methods (Coombes *et al.*, 1990), autoradiography (Reubi and Torhorst, 1989) and EGFR-associated phosphotyrosine kinase activity (Baugnet-Mahieu and Lemaire, 1990).

Ligand-binding assay is generally accepted as the 'gold standard' in EGFR measurements, though even here there is disagreement about the cut-off used to define positivity (Klijn *et al.*, 1992). Immunohistochemical assays (IHAs) are simple to perform, can be semiquantitative and have the advantage over LBA of showing the tissue distribution of EGFR. However, there are two major problems with the IHAs described in the literature. Firstly, the majority do not work on paraffin-embedded material and so cannot be applied to archival material. Secondly, few studies have attempted to validate the assays used. In addition some anti-EGFR antibodies are raised against receptor in glycosylated form. These will potentially cross-react with blood group antigens and make interpretation of results difficult (Gerdin *et al.*, 1992). Care must therefore be taken to ensure that the antibody used (in any assay method) is raised against epitopes on the EGFR protein and not on associated carbohydrate moieties.

We have developed an immunohistochemical assay for EGFR which works on paraffin-embedded sections and have validated this on a tumour set for which EGFR had previously been measured by ligand-binding assay. To find a clinically relevant cut-off point for this assay, analysis of the prognostic significance was determined for all possible values. The expected relationship with ER status was also assessed.

### Materials and methods

### *Patient selection*

Eighty-eight cases of previously untreated primary operable breast cancer were selected from the database at the John

Correspondence: JC Newby
Received 20 September 1994; revised 26 January 1995; accepted 7 February 1995

Radcliffe Hospital from 1989 to 1991. The criteria for selection were that EGFR had been previously measured by ligand-binding assay (Nicholson *et al.*, 1988*b*) and that oestrogen receptor (ER) status was known. Five micron paraffin sections from a representative block of each case were used for immunostaining.

Information on patient age, tumour size, adjuvant therapy and clinical course was taken from the breast cancer patient database. The median age of these patients was 55 years (range 28–83 years). Tumours ranged in size from 1.0 to 8.0 cm (mean 2.5 cm). In six cases nodal status was not known. Of the remaining 82, 43 were node positive (52%). Sixty per cent of cases were ER positive ($\geqslant 5$ fmol mg$^{-1}$ protein). Of the 88 patients, 26 received no adjuvant systemic therapy, 23 received chemotherapy alone, 35 received hormone therapy alone (33 tamoxifen, two aminoglutethimide), two received both and for two this information was not available. Follow-up was at 3 month intervals, with a median length of 39 months (range 17–51 months).

### *EGFR immunohistochemical assay*

The antibody used in this assay was a mouse monoclonal, subclass IgG1, raised against purified, denatured EGFR, and kindly provided by Biogenex, CA, USA (Cat. No. MU-207-UC). It reacts with a polypeptide epitope of the external domain of the molecule and does not interfere with EGF binding. A series of preliminary experiments were conducted to optimise the staining procedure. The final conditions selected were as follows: tissue was routinely fixed in formalin and then embedded in paraffin wax blocks. Five micron sections from the blocks were dewaxed in xylene, then hydrated through graded alcohols to water. After washing in water, endogenous peroxidase activity was blocked using a 3% solution of hydrogen peroxide in distilled water for 15 min at room temperature. Slides were washed then taken to phosphate-buffered saline (PBS) at 37°C for 15 min, followed by digestion in pronase (Sigma) at 0.05% (w/v) in PBS for 15 min at 37°C. After washing in PBS, non-specific binding was blocked using normal rabbit serum (Dako) diluted 1:5 in PBS. This was drained, primary antibody (MU207) applied at a dilution of 1:10 in PBS and the slides were then incubated at room temperature overnight. Following washing in PBS, secondary antibody (biotinylated rabbit anti-mouse IgG; Dako) was added and incubated for 1 h at room temperature. Slides were washed again and streptavidin–biotin–horseradish peroxidase complex (Dako) applied and incubated for 30 min at room temperature. Slides were washed again and developed in 0.05% 3,3'-diaminobenzidine solution with hydrogen peroxide. Sections were counterstained with Mayer's haematoxylin. Control slides of known EGFR-positive and -negative breast cancers were included in each staining run.

Slides were scored for percentage malignant cells showing membrane staining, averaged across ten high-power fields, and for overall intensity of staining on a scale of 0–3 (0 being no staining, 1 weak staining, 2 moderate staining and 3 strongly positive staining). The two scores were then multiplied to give the final result (possible range 0–300). A score of $\geqslant 35$ was counted as positive (see Results). The staining and scoring for EGFR were performed without knowledge of any other data [e.g. ER, EGFR (LBA), clinical outcome]. The preliminary assay optimisation experiments indicated that although staining for EGFR was heterogeneous within any one section, scores obtained by this method were similar for different sections of a tumour, and even for initial diagnostic biopsy and surgical excision specimens from the same patient.

### *EGFR ligand-binding assay*

This was performed as previously described (Nicholson *et al.*, 1988*b*). Results are expressed as fmol mg$^{-1}$ protein; a value of $\geqslant 20$ fmol mg$^{-1}$ protein is the cut-off used for this assay in routine practice in Oxford.

### *ER assay*

This was measured by the dextran-coated charcoal method (EORTC, 1980). Tumours with $\geqslant 5$ fmol mg$^{-1}$ cytosolic protein were considered positive.

### Results

### *EGFR immunostaining*

The pattern of staining in these cases largely conformed to known EGFR distribution. In normal tissues within the sections, skin epithelial cells were positive, with the strongest staining being in the basal layer cells. In benign breast tissues, both luminal and myoepithelial cells stained, with myoepithelial cells generally stronger. Stromal fibroblasts were weakly positive in some areas, both normal and malignant. Inflammatory cells were negative, with the exception of occasional plasma cells, in which intense cytoplasmic staining was seen, and some foamy macrophages in areas of duct ectasia which showed weak membrane staining. Smooth muscle in blood vessel walls stained, as did nerve sheath cells.

Within the breast cancers, when positive, malignant epithelial cells showed clear membrane staining (Figure 1). This membrane staining was generally heterogenous throughout the tumour. In addition, in a small number of cases there were some linear streaks of positive staining in the stroma surrounding nests of tumour cells. This was interpreted as representing remnants of normal tissue disrupted by malignant expansion. Only cases in which clear membrane staining of malignant epithelial cells was seen were counted as positive and scored. In a number of cases foci of benign breast epithelium staining positively for EGFR were found within tumours which were negative for EGFR (Figure 2).

### *Definition of positivity for EGFR*

A discriminatory value for EGFR(IHA) positivity was optimised by continuous testing across the range of all possible values in relation to prognosis (relapse-free and overall survival). The value selected was that which was associated with the greatest statistical significance for the comparison between the two groups it defined. For EGFR(IHA) this gave an optimal cut-off of 35, with 22% of the cases defined as positive. This compared with 44% of cases positive for EGFR(LBA) using the conventional cut-off of $\geqslant 20$ fmol mg$^{-1}$ protein.

Optimising the cut-off for the EGFR(LBA) data gave a cut-off value of approximately 80 fmol mg$^{-1}$, defining only 12% of cases as positive. This was too small a group on which to perform meaningful survival analysis. To allow a valid comparison between the prognostic significance of the two methods, an alternative approach was taken: a cut-off for EGFR(LBA) which defined the same proportion (22%) of cases as positive as the optimised EGFR(IHA) was found. This gave a cut-off of 38 fmol mg$^{-1}$.

**Figure 1** Breast carcinoma showing strongly positive staining for EGFR (IHA score = 300). Bar = 50μm.

**Figure 2** EGFR(IHA)-negative tumour with admixed EGFR-positive benign breast epithelium. Bar = 50μm.

**Figure 3** Linear correlation between EGFR(IHA) and EGFR(LBA) [cut-offs marked: - - - - - - , EGFR(IHA); - - - - = EGFR(LBA-20); — — — = EGFR(LBA-38)].

The different cut-offs are referred to below as EGFR(LBA-20) and EGFR(LBA-38). For all analyses the optimised cut-off of 35 is used for EGFR(IHA).

*Correlation with ligand-binding assay results*

Of the 19/88 (22%) cases which showed positive immunostaining of malignant epithelial cells, 14/19 (74%) were also positive on ligand-binding assay (LBA-20). Of those negative on immunostaining, 44/69 (64%) were also negative on ligand-binding assay. Overall there was agreement between EGFR as assessed by immunostaining (EGFR-IHA) and on ligand-binding assay (EGFR-LBA-20) in 66% of cases ($P = 0.004$). On a simple linear regression plot (Figure 3), it can be seen that the majority of the cases with discordant results were negative on immunostaining but positive to a variable degree on LBA. It is probably significant that, of the 25 cases which were negative for EGFR (IHA) but positive for EGFR(LBA-20), 16 sections also contained some benign breast epithelium, and in 14 of these 16 cases the benign epithelial elements were positive for EGFR. The intensity of staining in benign breast epithelium was similar to that seen in malignant epithelium. When the optimised LBA cut-off (LBA-38) was applied, the concordance increased to 82% ($P < 0.0001$). It can be seen from Figure 3 that the improved concordance using EGFR (LBA-38) is largely due to the loss from the relationship of cases which were IHA negative but LBA-20 positive.

*Association with other parameters*

No association was found between EGFR measured by either method and patient age or tumour size. Table I shows the number of EGFR-positive cases divided according to number of nodes involved for both assay methods. The expected inverse relationship with ER exists for EGFR as measured by IHA ($\chi^2 = 24.9$, $P < 0.0001$) and for EGFR (LBA-38)($\chi^2 = 8.3$, $P = 0.0071$), though it is not significant for EGFR (LBA-20) (Table I). Only two tumours were positive for both EGFR (IHA) and ER.

*Prognostic value*

Table II shows the results of univariate analysis of prognostic factors for this set of patients. It can be seen that, while nodal status was the most powerful prognostic factor of those analysed, size ≥2.5 cm ($P = 0.01$), ER status ($P = 0.05$) and EGFR (IHA) ($P = 0.02$) were all significant

**Table I** Relationship between ER and EGFR

| | *EGFR(IHA)* | | *EGFR(LBA-20)* | | *EGFR(LBA-38)* | |
|---|---|---|---|---|---|---|
| *ER* | *− ve* | *+ ve* | *− ve* | *+ ve* | *− ve* | *+ ve* |
| − ve | 18 | 17 | 15 | 20 | 22 | 13 |
| + ve | 51 | 2 | 34 | 19 | 47 | 6 |
| | $\chi^2 = 24.9$, $P < 0.0001$ | | Not significant | | $\chi^2 = 8.3$, $P = 0.0071$ | |

**Table II** Univariate analysis of prognostic factors for relapse-free (RFS) and overall (OS) survival

| | *RFS* | | *OS* | |
|---|---|---|---|---|
| | *Hazard ratio* | *p-value* | *Hazard ratio* | *p-value* |
| EGFR(IHA) | 2.38 (1.11–5.12)[a] | 0.02 | 2.60 (1.09–6.22) | 0.03 |
| EGFR(LBA-20) | 1.15 (0.56–2.35) | NS | 1.09 (0.47–2.53) | NS |
| EGFR(LBA-38) | 1.82 (0.83–3.99) | NS | 2.13 (0.87–5.24) | NS |
| Age (≥55 years) | 1.22 (0.60–2.50) | NS | 1.35 (0.58–3.12) | NS |
| Size (≥2.5 cm) | 2.51 (1.17–5.37) | 0.01 | 1.41 (0.58–3.39) | NS |
| ER | 0.49 (0.24–1.00) | 0.05 | 0.77 (0.33–1.79) | NS |
| No. of nodes | | | | |
| 0 | 1.00 | | 1.00 | |
| 1–3 | 2.39 (1.47–3.87) | <0.001 | 3.35 (1.83–6.13) | <0.001 |
| ≥4 | 5.70 (3.52–9.24) | | 11.21 (6.12–20.53) | |

[a]Figures in brackets = 95% confidence intervals. NS, not significant.

**Figure 4** Survival curves for relapse-free (**a**) and overall survival (**b**) stratified by EGFR(IHA). ———, EGFR < 35; - - - , EGFR ≥ 35.

for relapse-free survival (RFS), and EGFR (IHA) ($P = 0.03$) was the only other significant factor for overall survival (OS). EGFR (LBA-20 or -38) was not significant in either category. Figure 4 shows the survival curves for RFS and OS stratified by EGFR(IHA) (log-rank test and Kaplan–Meier product-limit method). Of the 88 patients, 23 had died during the follow-up period (15 node positive); ten had suffered recurrence but were still alive (seven node positive) and 55 remained disease free.

In multivariate analysis, using the Cox regression model and including all of the factors significant in univariate analysis, nodal status ($P < 0.001$) and ER status ($P < 0.01$) were independent factors for RFS; only nodal status was an independent prognostic factor for OS ($P < 0.001$).

### Discussion

Since Sainsbury *et al.*, first described EGFR as a prognostic factor in human breast cancer in 1985 (using a ligand-binding assay method), there have been a large number of studies which have measured EGFR in breast cancers by a variety of methods and with differing definitions and thus proportions of positivity. Grimaux *et al.* (1989) did not find EGFR (measured by LBA) to be of prognostic significance in a group of 68 node-positive cases but used a cut-off of 5 fmol $mg^{-1}$ protein rather than 10 fmol $mg^{-1}$, which was more widely used. Using 10 fmol $mg^{-1}$ as a cut-off, Spyratos *et al.* (1990) again did not find EGFR to have prognostic significance in either node-positive or node-negative patients. Other studies have agreed with Sainsbury's findings (e.g. Costa *et al.*, 1988, using LBA and Lewis *et al.*, 1990, using IHA on frozen sections).

Ligand-binding assay is the most widely applied method and is the current 'gold standard' for measuring EGFR. Immunohistochemical assays have the general advantages of being quick and simple to perform, requiring little material and showing the tissue distribution of the antigen concerned. Some studies have compared the results of LBA and IHA assays for EGFR. In their original study, Sainsbury *et al.* (1985) found that results obtained by immunostaining with the EGFR-1 antibody on frozen sections 'correlated with' the results of ligand-binding assay. In a more formal comparison, Toi *et al.* (1989) found a 94% concordance between immunostaining with the EGFR-1 antibody and results of ligand-binding assay. Using the same antibody in ovarian carcinomas, a concordance of 67% was found between the two methods (Owens *et al.*, 1992). Using a different antibody, MAb 425, on breast cancers, no significant differences were seen when EGFR which had been measured both biochemically and immunohistochemically was independently correlated with other tumour characteristics, but this study did not directly compare the two assay results (Beckman *et al.*, 1993). All of the above immunohistochemical studies were performed on frozen sections. A recent study has compared LBA, EIA and IHA and found reasonable agreement between the results of the three methods (72% concordance between IHA and LBA) (Iwase *et al.*, 1993). That study concluded that EIA is the most appropriate method for use with clinical samples on the basis that it had the strongest prognostic value in the patient group examined. None of the IHAs described in these studies were conducted on paraffin-embedded sections, a procedure which has been difficult to perform successfully for EGFR. The availability of such an assay would be extremely valuable in the investigation of the biological/clinical significance of EGFR in the enormous stores of archival pathological material.

We have described and validated an immunohistochemical assay for EGFR which works on formalin-fixed, paraffin wax-embedded sections. This employs a monoclonal antibody which is raised against purified EGFR and does not cross-react with blood group antigens. This cross-reaction has been shown to be a problem with some previously described immunohistochemical assays for EGFR (Gerdin *et al.*, 1992). The pattern of staining produced by this assay is consistent with the known distribution of EGFR in breast tissues, both benign and malignant, and also in other normal components of these sections such as skin, nerves and smooth muscle (Damjanov *et al.*, 1986). It has the significant advantage over LBA/EIA of showing the tissue distribution of the EGFR, eliminating the problem of possible confounding of results by expression in normal breast epithelium (and other tissue elements). Benign breast epithelium has been previously shown to express higher mean levels of EGFR than malignant breast epithelium by both LBA and immunohistochemistry on frozen material (Travers *et al.*, 1988; Barker *et al.*, 1989; Dittadi *et al.*, 1993).

The proportion of tumours staining positively by this IHA is relatively low (22%). This is at the lower end of the range of positivity rates found in other studies (14–91% for a variety of assay methods, 14–65% for immunohistochemical analyses using the EGFR1 antibody on frozen tissue; Klijn *et al.*, 1992). Our positivity rate may be within the expected variability range found when comparing results across several often relatively small studies. Alternatively, it may relate to the assay method in which antigen retrieval in the form of protease digestion is essential. The length of the digestion phase of the protocol is limited by the need to preserve tissue morphology. It is possible therefore that the sensitivity of the assay is reduced relative to other methods as one may be unable to 'retrieve' all antigen present while still preserving tissue morphology.

Measurement of EGFR using this immunohistochemical assay shows a positive, but by no means perfect, correlation with the ligand-binding assay, and as described above there are good reasons for arguing that LBA, or any of the other methods which use homogenates of tissue, are not the most appropriate methods for assessing EGFR in clinical material. The majority of discordant results are those which are negative by immunostaining but positive by LBA. The majority of these have positive-staining benign breast epithelium. While these paraffin sections are not contiguous with the portions of tumour used in the LBA, it does provide a probable explanation for the majority of discordant results. This immunohistochemical assay may therefore be more appropriate for use with clinical specimens.

The numbers of patients investigated is too small for a formal study of prognosis, and the optimisation of cut-off for the IHA not only inevitably led to it having prognostic significance, but also has statistical problems (Altman *et al*., 1994). All the same, it was felt that the potential clinical usefulness of this approach to EGFR measurement could be preliminarily assessed by comparing its prognostic significance and relationship with other tumour parameters in this group of patients with those of the ligand-binding assay results. The intention was to compare the value of an established method with a new method in this cohort of limited size. When compared with the LBA, EGFR (IHA) showed the expected inverse association with ER while EGFR (LBA-20) did not (Table I). EGFR (LBA-38) did show a significant inverse correlation, but the relationship was not as strong as that with the IHA ($\chi^2 = 8.3$ *vs* $\chi^2 = 24.9$). No association was seen between nodal status and EGFR measured by either method. In terms of prognosis, (Table II) EGFR (IHA) was a significant factor (on univariate analysis) for both RFS and OS and retained significance on multivariate analysis for RFS ($P < 0.01$). EGFR (LBA-20 or -38) was not a significant prognostic factor for either RFS or OS.

The data on comparability are thus very encouraging, indicating that the IHA is likely to be at least as useful as the LBA for clinical assessment of EGFR. The statistically stronger relationships with EGFR(IHA) may be due to the exclusion by this approach of EGFR-expressing normal benign tissue, which would be expected to be biologically less relevant (or entirely irrelevant) in relation to disease progression. Confirmation of the prognostic significance of EGFR(IHA) would obviously require a much larger patient group. Such a study is planned for the near future.

In conclusion, we have developed and validated an immunohistochemical assay for EGFR which is quick and simple to perform, requires small amounts of tissue and can be applied to paraffin-embedded sections and thus to archival material.

**Acknowledgements**

We are extremely grateful to Dr Atul Tandon and Biogenex, California, for kindly providing the anti-EGFR antibody used in this study. We would also like to thank Paul Newcomb and Paul Savage for performing EGFR ligand-binding assays. The Royal Marsden Hospital Committee for Clinical Research supported Dr JC Newby for this work.

### References

ALTMAN DG, LAUSEN B, SAUERBREI W AND SCHUMACHER M. (1994). Dangers of using 'optimal' cutpoints in the evaluation of prognostic factors. *J. Natl Cancer Inst.*, **86**, 829-834.

BARKER S, PANAHY C, PUDDEFOOT JR, GOODE AW AND VINSON GP. (1989). Epidermal growth factor receptor and oestrogen receptors in the non-malignant part of the cancerous breast. *Br. J. Cancer*, **60**, 673-677.

BAUGNET-MAHIEU L AND LEMAIRE M. (1990). Expression of epidermal growth factor receptor and c-erb-B2 oncoprotein in human tumours (abstract 138). *Eur. J. Cancer*, **26**, 181.

BECKMANN MW, TUTSCHEK B, KRUGER KH, NIEDERACHER D, RISSE BC, RUPPERT C, SCHNURCH H-G AND BENDRER HG. (1993). Biochemical and immunohistochemical detection of the epidermal growth factor receptor in breast tumour specimens. *Int. J. Oncol.*, **3**, 389-397.

CARRAWAY KL AND CANTLEY LC. (1994). A Neu acquaintance for ErbB3 and ErbB4: a role for receptor heterodimerisation in growth signalling. *Cell*, **78**, 5-8.

COOMBES RC, BARRETT-LEE PJ AND LUQMANI YA. (1990). Growth factor expression in breast tissue. *J. Steriod Biochem. Mol. Biol.*, **37**, 833-836.

COSTA S, STAMM H, ALMENDRAL A, LUDWIG H, WYSS R, FABBRO D, ERNST A, TAKAHASHI A AND EPPENBERGER V. (1988). Predictive value of epidermal growth factor receptor in breast cancer. *Lancet*, **ii**, 1258.

DAMJANOV I, MILDNER B AND KNOWLES BB. (1986). Immunohistochemical localisation of the epidermal growth factor receptor in normal human tissues. *Lab. Invest.*, **55**, 588-89.

DITTADI R, DONISI PM, BRAZZALE A, CAPELLOZZA L, BRUSCAGNIN G AND GION M. (1993). Epidermal growth factor receptor in breast cancer. Comparison with non-malignant breast tissue. *Br. J. Cancer*, **67**, 7-9.

EORTC BREAST COOPERATIVE GROUP. (1980). Revision of the standards for the assessment of hormone receptors in human breast cancer: Report of the second EORTC workshop, held on 16-17 March 1979 in the Netherlands Cancer Institute. *Eur. J. Cancer*, **16**, 1513-1515.

FOEKENS JA, PORTENGEN H, VAN PUTTEN WLJ, TRAPMAN AMAC, REUBI JC, ALEXIEVA-FIGUSCH J AND KLIJN JGM. (1989). Prognostic value of receptors for IGF-1, somatostatin and EGF in human breast cancer. *Cancer Res.*, **49**, 7002-7009.

FOX SB, SMITH K, HOLLYER J, GREENALL M, HASTRICH D AND HARRIS AL. (1994). The epidermal growth factor receptor as a prognostic marker: results of 370 patients and review of 3009 patients. *Breast Cancer Res. Treat.*, **29**, 41-49.

GASPARINI G, BEVILACQUA P, POZZA F, MELI S, BORACI HP, MARUBINI E AND SAINSBURY JRC. (1992). Value of epidermal growth factor receptor status compared with growth fraction and other factors for prognosis in early breast cancer. *Br. J. Cancer*, **66**, 670-676.

GERDIN E, JUHLIN C, MALMGREN M AND GERDIN B. (1992). Immunohistochemical identification of receptors for epidermal growth factor in tumour endothelium may be affected by cross-reactivity to blood group A antigen. *Anat. Pathol.*, **99**, 28-31.

GRIMAUX M, ROMAIN S, REMVIKOS Y, MARTIN PM, AND MAGDELENAT H. (1989). Prognostic value of epidermal growth factor receptor in node-positive breast cancer. *Breast Cancer Res. Treat.*, **14**, 77-90.

HARRIS AL, NICHOLSON S, SAINSBURY JRC, FARNDON JR AND WRIGHT C. (1989). Epidermal growth factor receptor in breast cancer: association with early relapse and death, poor response to hormones and interactions with neu. *J. Steroid Biochem.*, **34**, 123-32.

IWASE H, KOBAYASHI S, ITOH Y, KUZUSHIMA T, YAMASHITA H, IWATA H, NAITO A, YAMASHITA T, ITOH K AND MASAOKA A. (1993). Clinical value of enzyme-immunoassay of epidermal growth factor receptor in human breast cancer. *Breast Cancer Res. Treat.*, **28**, 215-21.

KLIJN JGM, BERNS PMJJ, SCHMITZ PIM AND FOEKENS JA. (1992). The clinical significance of epidermal growth factor receptor in human breast cancer: review of 5232 patients. *Endocrine Rev.*, **13**, 3-17.

KOENDERS PG, BEEX LVAM, KIENHUIS CBM, KLOPPENBERG PWC AND BENRAAD TLJ. (1993). Epidermal growth factor receptor and prognosis in breast cancer. *Breast Cancer Res. Treat.*, **25**, 21-27.

LEWIS S, LOCKER A, TODD JH, BELL JA, NICHOLSON R, ELSTON CW, BLAMEY RW AND ELLIS IO. (1990). Expression of epidermal growth factor receptor in breast carcinomas. *J. Clin. Pathol.*, **43**, 385-89.

MURRAY PA, BARRETT-LEE P, TRAVERS M, LUQMANI Y, POWLES T, AND COOMBES RC. (1993). The prognostic significance of transforming growth factors in human breast cancer. *Br. J. Cancer*, **67**, 1408-1412.

NICHOLSON RI, McCLELLAND RA, GEE JMW, MANNING DL, CANNON P, ROBERTSON JFR, ELLIS IO AND BLAMEY RW. (1994). Epidermal growth factor receptor expression in breast cancer: association with response to endocrine therapy. *Breast Cancer Res. Treat.*, **29**, 117-125.

NICHOLSON S, HALCROW P, SAINSBURY JRC, ANGUS B, CHAMBERS P, FARNDON J AND HARRIS AL. (1988*a*). Epidermal growth factor receptor status associated with failure of primary endocrine therapy in elderly post-menopausal patients with breast cancer. *Br. J. Cancer*, **58**, 810-814.

NICHOLSON S, SAINSBURY JRC, NEEDHAM GK, CHAMBERS P, FARNDON JR AND HARRIS AL. (1988*b*). Quantitative assays of epidermal growth factor receptor in human breast cancer: cut-off points of clinical relevance. *Int. J. Cancer*, **42**, 36-41.

NICHOLSON S, SAINSBURY JRC, HALCROW P, KELLY P, ANGUS B, WRIGHT C, HENRY J, FARNDON JR AND HARRIS AL. (1991). Epidermal growth factor receptor: results of a 6 year follow up study in operable breast cancer with emphasis on the node negative sub group. *Br. J. Cancer*, **73**, 146–51.

NORMANNO N, CIARDELLO F, BRANDT R AND SALOMON DS. (1994). Epidermal growth factor related peptides in the pathogenesis of human breast cancer. *Breast Cancer Res. Treat.*, **29**, 11–27.

OWENS OJ, STEWART C, LEAKE RE AND McNICOL AM. (1992). A comparison of biochemical and immunohistochemical assessment of epidermal growth factor receptor expression in ovarian cancer. *Anticancer Res.*, **12**, 1455–1458.

PARKER PJ, YOUNG S, GULLICK WJ, MAYES ELV, BENNETT P AND WATERFIELD M. (1984). Monoclonal antibodies against the human epidermal growth factor receptor from A431 cells. *Biol. Chem.*, **259**, 9906–9912.

RAJKUMAR T AND GULLICK WJ. (1994). The type 1 growth factor receptors in human breast cancer. *Breast Cancer Res. Treat.*, **29**, 3–9.

REUBI JC AND TORHORST J. (1989). The relationship between somatostatin epidermal growth factor and steroid hormone receptors in breast cancer. *Cancer*, **64**, 1254–1260.

SAINSBURY JRC, FARNDON JR, SHERBET VG AND HARRIS AL. (1985). Epidermal growth factor receptors and oestrogen receptors in human breast cancer. *Lancet*, **i**, 364–366.

SAINSBURY JRC, FARNDON JR, NEEDHAM GK, MALCOLM AJ AND HARRIS AL. (1987). Epidermal growth factor receptor status as predictor of early recurrence and death from breast cancer. *Lancet*, **i**, 1398–1402.

SPYRATOS F, DELARUE JC, ANDRIEU C, LIDEREAU R, CHAMPEME MH, HACENE K AND BRUNET M. (1990). Epidermal growth factor receptors and prognosis in primary breast cancer. *Breast Cancer Res. Treat.*, **17**, 83–89.

TOI M, HAMADA Y, NAKAMURA T, MUKAIDA H, SUEHIROS S, WADA T, TOGE T, NIMOTO M AND HATTORI T. (1989). Immunocytochemical and biochemical analysis of epidermal growth factor receptor expression in human breast cancer tissues: relationship to oestrogen receptor and lymphatic invasion. *Int. J. Cancer*, **43**, 220–225.

TOI M, AKIHKO O, YAMADA H AND TOGE T. (1991). Epidermal growth factor receptor expression as a prognostic indicator in breast cancer. *Eur. J. Cancer*, **27**, 977–980.

TRAVERS M, BARRETT-LEE P, BERGER V, LUQMANI Y, GAZET JC, POWLES TJ AND COOMBES RC. (1988). Growth factor expression in normal, benign and malignant breast tissue. *Br. Med. J.*, **296**, 1621–1624.